# From Open to MIS, evolving practice for posterior approaches in high-energy thoracolumbar vertebral fractures: a retrospective cohort study

**DOI:** 10.1016/j.bas.2026.106002

**Published:** 2026-03-03

**Authors:** Simone Grannò, Barbara Vukic, Francesca Brigatti, Eva Cochard, Adrien May, Granit Molliqaj, Nicolas Lauper, Dennis E. Dominguez, Karl Schaller, Enrico Tessitore

**Affiliations:** a Division of Neurosurgery, Department of Clinical Neurosciences, Geneva University Hospitals, Geneva, Switzerland; b Spine Team, Division of Orthopedic Surgery and Musculoskeletal Trauma Care, Geneva University Hospitals and University of Geneva's Faculty of Medicine, Rue Gabriel-Perret-Gentil 4, CH-1205, Geneva, Switzerland

**Keywords:** Thoracolumbar fractures, Minimally invasive surgery, Wiltse approach, Percutaneous fixation, Vertebral augmentation

## Abstract

**Introduction:**

thoracolumbar fractures often require fixation. Minimally invasive surgery (MIS), including Wiltse and percutaneous techniques, aims to reduce morbidity compared to open surgery.

**Research question:**

to evaluate multidimensional outcomes in high-energy thoracolumbar fractures treated via MIS versus open surgery.

**Methods:**

a single-centre retrospective cohort study including 82 neurologically intact adults (43.2 ± 18.5 y) who underwent open or MIS posterior fixation. Patients were divided into the Open (*n* = 23), versus MIS Wiltse (*n* = 30) and Percutaneous (*n* = 29) cohorts. The primary outcome was length of stay. Secondary outcomes included intraoperative blood loss, radiographic correction, second-stage surgery, concomitant kyphoplasty, pain and return-to-work status.

**Results:**

Length of stay was reduced for Wiltse (11.2 ± 4.5 *d*, *p* < 0.001) and Percutaneous (16.5 ± 7.8 *d*; *p<*0.05) versus Open patients (21.6 ± 10 *d*). Return-to-work delay was shorter in Wiltse (34 ± 12 *d*; *p* < 0.01) versus Open patients (72 ± 48 *d*). Intraoperative estimated blood loss was significantly reduced in Wiltse (929.5 ± 453.9 ml; *p* < 0.003) and Percutaneous (653 ± 429 ml; *p* < 0.0001) compared to Open surgery (1584 ± 1027 ml). This was mirrored in peri-operative ΔHb (37 g/L Open, 25 g/L Wiltse, *p* < 0.05; 17 g/L Percutaneous, *p* < 0.001). Pain recovery was faster in MIS. Second-stage corpectomy was performed in 47.8% (Open), 3.3% (Wiltse) and 33.2% (Percutaneous). Kyphoplasty was more frequent in Wiltse (66.6%) than Percutaneous (31%). Radiographic correction was maintained in all cohorts.

**Conclusions:**

MIS approaches were associated with comparable fracture stabilisation to open surgery, with shorter hospital stay and lower estimated blood loss.

## Highlights

• MIS reduced length of stay.
• Estimated blood loss was significantly lower in MIS vs. Open approaches.
• Second-stage corpectomy was less frequent with MIS.
• All approaches showed stable fracture correction over follow-up.
• Pain recovery was faster in MIS cohorts.

## 1 Introduction

Thoracolumbar vertebral fractures, primarily resulting from high-energy trauma (e.g., falls, motor vehicle accidents), are the most common spinal injuries, leading to varying levels of neurological impairment, disability, and chronic pain across age groups (Zileli et al., 2021). Around 25% of these fractures involve spinal cord injury (Katsuura et al., 2016), severely impacting patients’ health and quality of life (Van den Berg et al., 2010), highlighting the importance of effective management strategies in both neurosurgery and orthopaedic spine surgery (Lan et al., 2017; Kim et al., 2011).

The thoracolumbar junction, where the rigid thoracic spine transitions to the mobile lumbar spine, is anatomically and biomechanically vulnerable. This region also marks the transition of the spinal cord (CNS) into the cauda equina roots (PNS), resulting in complex neurological consequences in cases of injury. The AO Spine/Magerl classification system, commonly used in clinical practice, helps determine the need for conservative or surgical intervention based on injury mechanisms and the involvement of the posterior osteoligamentous complex (Schnake et al., 2017; Divi et al., 2019).

Operative treatment is a cornerstone in the management of these fractures, particularly for cases involving segmental instability, significant deformity, or neurological deficit (Wood et al., 2014).

Historically, the treatment of thoracolumbar fractures centred on fusion surgery, relying on large incisions and bone graft to achieve arthrodesis. Over the last few decades, however, a paradigm shift has occurred toward non-fusion approaches, recognising that many fractures can be successfully managed without the morbidity associated with extended fusion constructs. In such cases, pedicle screw fixation is used to stabilise the injured segments while preserving potential motion and limiting unnecessary bone removal. Additionally, in case of significant vertebral body deformation, corpectomy or disk cages, as well as augmentation devices can be placed to restore vertebral body height without the need for graft-based fusion. This evolving practice acknowledges that, in properly selected patients, strong instrumentation can be sufficient for fracture healing and pain control, thereby reducing operative invasiveness, morbidity, and the duration of recovery. As a result, non-fusion surgery has become an increasingly accepted strategy that redefines the balance between mechanical stability and physiological motion preservation. Different techniques for pedicle screw fixation are available, each offering distinct benefits and challenges.

The traditional “open” approach involves a large midline incision and detachment of paraspinal muscles, providing direct visualisation of the anatomy for free-hand screw placement but often causing significant bleeding and delayed recovery (Zhu et al., 2023). In contrast, the paraspinal sacrospinalis-splitting (Wiltse) approach, developed by Dr. Leon Wiltse in the 1970s, uses blunt dissection along the natural separation of the multifidus and longissimus muscles via paramedian or single midline incisions. This technique reduces intraoperative bleeding and preserves paraspinal musculature, lowering muscle atrophy (Junhui et al., 2017) and postoperative pain (David et al., 2019). It is considered under the umbrella term of minimally invasive surgery (MIS).

The percutaneous approach, also classified as MIS, uses paramedian keyhole incisions with CT-guided 3D navigation for pedicle screw placement. Studies suggest it reduces muscle dissection, postoperative pain, and hospital stay (Zhu et al., 2023; Camacho et al., 2019). However, MIS is highly operator-dependent, with a steep learning curve and a significant rate of screw misplacement, especially in complex fractures (Jiang et al., 2022). Overall, current evidence suggests that the benefit of MIS approaches also tends to sharply decrease in more complex fractures. Both Wiltse and percutaneous approaches are now widely employed, not only in trauma but also in degenerative spine surgery (David et al., 2019; Dong et al., 2014; Du et al., 2017; Bauer et al., 2025).

Despite the extensive use of these surgical approaches, consensus on the optimal strategy in traumatic fractures remains elusive. This study thus aims to compare clinical outcomes for patients with high-energy thoracolumbar fractures treated by posterior fixation using these three techniques: Open, Wiltse, and Percutaneous.

To best isolate the specific effect of surgical approaches and minimize confounding factors, we selected a *neurologically intact* patient population, in line with previous studies (Jiang et al., 2022; Giordan et al., 2022). Patients presenting with neurological deficits typically require decompression surgery, which mandates at least a partially open exposure irrespective of the fixation strategy. Including such patients would therefore introduce unavoidable and substantial bias.

We thus evaluated differences in length of stay (hospitalisation), complication rates, intraoperative blood loss, pain management and radiological outcomes such as fracture correction over long-term follow-up. This single-center study highlights the collective expertise and evolving practices of our neurosurgical and orthopedic spinal teams, aiming to inform surgical decision-making, enhance patient outcomes, and guide future research.

## 2 Methods

### 2.1 Patient selection, consent and anonymisation

**Fig. 1:**
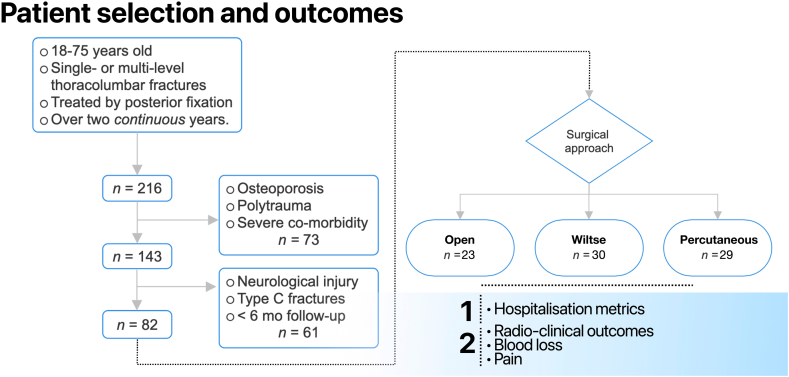
Patient selection flowchart, outlining inclusion and exclusion criteria.

The retrospective study protocol was submitted to the Business Administration System for Ethics Committees (BASEC) portal and examined by the *Commission Cantonale d'Ethique de la Recherche sur l'être humain* (CCER), receiving ethical approval in July 2025, code *CCER 2025-00378.* Electronic clinical records were consulted first searching for all vertebral fractures treated by posterior fixation over two years, either in 2010-2012 or 2021-2023 (Fig. 1, flowchart). We aimed to evaluate our department's transition from traditional open fixation to MIS techniques. As open fixation for the target patient population has not been performed in our institution for over a decade, an historical cohort was the only valid and ethically feasible comparator.

Patients were sequentially selected following the inclusion and exclusion criteria outlined in the results to assemble a retrospective database. All patients identifying details were anonymised using a random code generator and never included in the database. Patient consent to their clinical data being employed in this retrospective study was obtained via our standard institutional protocol. Specifically, patients had either signed a pre-operative general consent to research or, if this was not the case, they were contacted via both telephone and post to sign a consent form applicable only for this study.

### 2.2 Surgical strategy

A key aspect of this retrospective series lies in grouping our patient cohort on three distinct surgical strategies reflecting the evolution of our institutional practice towards MIS. They are centred around the concept of non-fusion surgery with or without vertebral augmentation based on fracture morphology and stability.

In this study, the “Open” cohort first underwent conventional open posterior stabilisation with a controlled reduction system (USS Fracture®). A second-stage index corpectomy was then performed if indicated for more severe or complex fracture patterns. The “Percutaneous” cohort, represents a shift in our treatment approach, whereby patients initially received minimally invasive fixation (CD-Horizon®, Expedium®), with concomitant SpineJack® augmentation or second-stage corpectomy as necessary. This approach aimed to minimize muscle dissection during the early phase while still providing the option for adequate vertebral reconstruction. Meanwhile, the “Wiltse” cohort underwent posterior stabilisation with a controlled reduction system (USS Fracture®) via a paraspinal muscle-splitting approach, often combined with SpineJack® when appropriate, thereby obviating the need for a staged corpectomy in most cases. Additionally, several patients in all groups underwent *in situ* fixation alone without vertebral augmentation. Collectively, these strategies reflect a shift toward single-stage, muscle-preserving, and biomechanically robust treatments for thoracolumbar fractures at our institution.

### 2.3 Data collection

For each patient, the outcomes and metrics outlined in the results section were selected *a priori*, after interrogation of the literature on similar studies, and then collected retrospectively by reviewing each electronic patient file individually. Overall, the following clinical, radiological and surgical metrics were collected: patient ID, operating team, surgical approach (*Open/Wiltse/Percutaneous*) age, sex, number of fractured levels, fracture level, AO Spine thoraco-lumbar spine injury classification system type, pre-op, post-op and at discharge fracture angle (*deg*), AOSpine neurological symptoms, Visual Analog Scale (VAS) pain levels at admission, VAS post op at 48hrs, VAS at discharge, pre-op imaging type, post-op imaging type, post-op imaging features, peri-op complications (*Y/N*), complication type, fixation level, SpineJack® kyphoplasty (Y/N), SpineJack® level, second-stage surgery (Y/N), operating time (*min*), fixation system employed, post-op fracture angle (deg), follow up fracture angle (*deg*), pre-op, post op at 24-48hrs Hemoglobin (Hb, *g/L*) and Hematocrit (Hct, %) levels, intervention delay (*d*), hospitalisation duration (*d*), rehabilitation (*Y/N*), rehabilitation duration (*d*), functional recovery/return-to-work status (*d*) follow-up duration (*w*), follow-up imaging features, long term complications (*Y/N*), complication type. Validated patient-reported outcome measures were not routinely recorded for acute trauma cases during the study periods. Therefore, functional recovery was assessed using clinically documented return-to-work status as a pragmatic surrogate endpoint. The database was assembled in Apple Numbers 14.2 and Microsoft Excel. All raw data are available upon reasonable request.

Patients were selected as having sustained high-velocity trauma, which was defined by high-energy mechanisms such as motor vehicle collisions, falls from heights greater than 1 m, or other impacts involving substantial acceleration or deceleration force. Fractures were defined according to number of fractured levels and AO Spine classification. Importantly, for all B-type fractures, when applicable the associated A subtype was noted, but the fractures were considered only based on their B-type (for example, a B2 + A2 fracture was classified as B2 for data analysis purposes). Therefore, the stated frequency of A-type fractures does not include those part of a B-type fracture.

Fracture angle kyphotic deformation was measured for each fracture, as the angle formed between the superior and the inferior endplate of the vertebra at the fractured level, on lateral standing radiographs or CT/MRI images where standing radiographs were not available or contraindicated. Comparative assessment of fracture angles over time was always measured in the same imaging sequence. Visual Analog Scale (VAS) pain intensity was quantified from 0 to 10 out of 10 based on clinical records. Peri-operative complications were defined as complications arising directly from the surgical procedure itself (eg.: hardware malposition, major intraoperative bleeding, surgical site infections) and did not include iatrogenic complications such as systemic infections of non-surgical origin or new comorbidity. Long term complications were defined as direct complications of the surgical procedure requiring further treatment (eg.: hardware displacement or fracture, severe mechanical pain) and not including minor chronic pain or elective hardware ablation after complete radiological recovery of the fracture. Functional recovery or “reurn-to-work” status, was quantified as duration of the period during which work or regular activities were interrupted by medical certificates, irrespective of actual employment. The term is employed to align with existing literature.

Estimated blood loss (EBL) was quantified based on previous literature in the field (Jiang et al., 2023), first employing Nadler's formula to estimate patient blood volume (PBV).

• Male = [0.3669 × (height, m)^3^ + 0.03219 × (weight, kg) + 0.6041]
• Female = [0.3561 × (height, m)^3^ + 0.03308 × (weight, kg) + 0.1833]

Then, the Gross equation for Maximum Allowable Blood Loss (ABL) was employed to yield EBL:

PBV (mL) × (Hct_pre_ − Hct_post_) / Hct_avg_

Where Hct = hematocrit (%)

### 2.4 Statistical analysis

All statistical analysis was performed in GraphPad Prism 10. For all quantitative variables, descriptive statistics were expressed as mean and standard deviation (SD). For all categorical variables, contingency analysis was performed by Chi-square test. Gaussian fit was performed by non-linear regression. All comparisons between two matched variables (eg pre-op and post-op Hb levels within one surgical cohort) were analysed by paired *t*-test. All comparisons between three matched variables (eg VAS at admission, post-op and follow-up) were analysed by repeated measures one-way ANOVA where all data points were available, or by fitting a mixed-effects model with restricted maximum likelihood (REML) for missing data points. All comparisons between three independent groups (eg surgical approach) were performed by one-way ANOVA. For all multiple comparisons, Tukey's *post-hoc* correction was employed. In all cases, statistical significance was defined as *p <* 0.05. All raw data are available upon reasonable request. Multi-level fractures where analysed as individual fractures when assessing fracture prevalence in each cohort and radiological correction. Conversely, when analysing kyphoplasty and corpectomy rates, they were grouped by individual patients, to better reflect patient-level intervention.

### 2.5 Figure design

All figures were designed in GraphPad Prism 10 and Apple Keynote 14.2. A colour scheme compatible with colour blindness was employed. For all figures with matched data, all individual data points were shown and paired. For all non-matched data, box-and-whiskers plots were employed, where the line inside the box represents the median, the box edge the interquartile range (25th to 75th) and the whiskers the minimum and maximum values.

### 2.6 Manuscript drafting and referencing

The manuscript was assembled in Apple Pages 14.2 and Microsoft Word. All references were indexed in EndNote 20.

## 3 Results

### 3.1 Inclusion criteria and patient population

We thus set out to retrospectively assess differential clinical outcomes in patients who underwent spinal fixation surgery for traumatic thoracolumbar fractures employing either a traditional “Open”, MIS “Wiltse” or fully “Percutaneous” approach. In order to identify the target population, we interrogated clinical records, refining the sample sequentially (Fig. 1, flowchart). The core inclusion criterion was patients aged 18-75 years old, who suffered single- or multi-level high-energy thoracolumbar fractures, treated surgically by posterior fixation, over two *continuous* years. This timeframe was selected to assess consistent clinical practice with the highest possible overlap of attending surgeon.

However, because of evolving local practices, we had *a priori* knowledge that patients were no longer treated by the open approach after 2013, corresponding to the widespread introduction of the SpineJack® Implantable Fracture Reduction System (Stryker) in our practice. Therefore, records were interrogated for patients treated in 2010-2012 via the Open approach, and 2021-2023 for the MIS approaches.

**Fig. 2:**
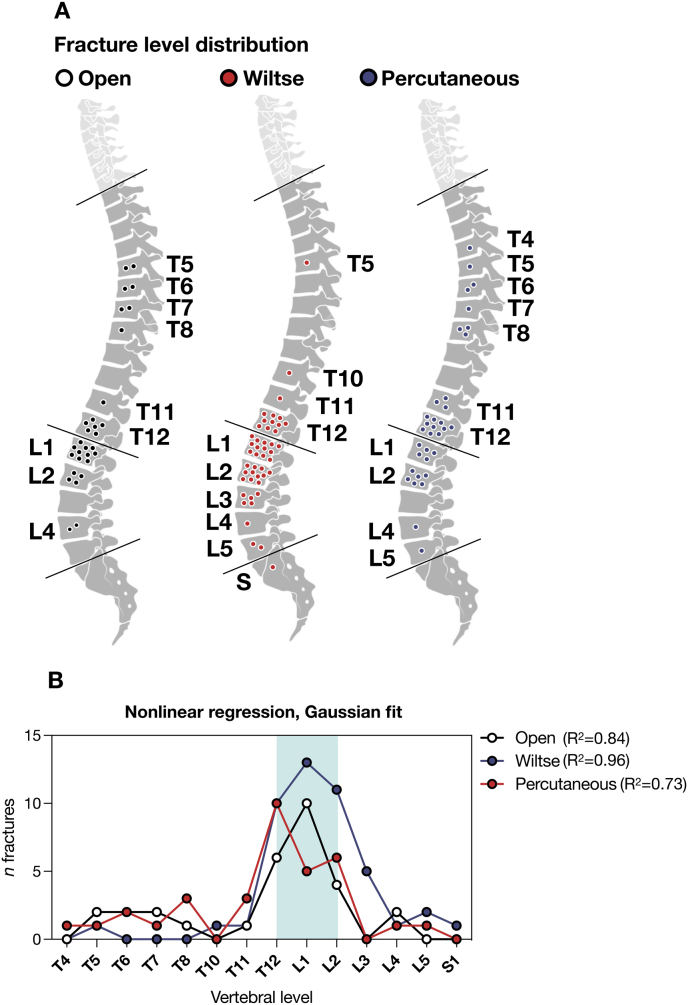
Fracture level overview. **A** - Visual summary of overall location of fractures treated by Open (white), Wiltse (red), or Percutaneous (blue) approaches. **B** - Fracture frequency in each cohort as a function of vertebral level, showing good fit for a Gaussian distribution around the thoraco-lumbar junction.

Exclusion criteria were severe co-morbidity, polytrauma including concomitant cervical fractures, non-vertebral bony or organic lesions, osteoporotic and low-energy fracture, type C translation injuries according to the AOSpine classification system, neurologic injury requiring decompression surgery according to the AOSpine classification system (N2-N4), less than 6 months follow up. This approach yielded a sample of *n=*82 patients, for a total of *n=*109 fractures (Table 1). Operative reports were consulted for each case to stratify patients into three groups by surgical approach. This yielded the “Open” (*n=*23, *n=*32 fractures), “Wiltse” (*n=*30, *n=*42 fractures) and “Percutaneous” (*n=*29, *n=*35 fractures) cohorts (Fig. 1, Fig. 2).

**Table 1:** **A -** Overall cohort characteristics. **B** – Outcomes ns - non significant; ∗^# - specific *p* values for direct comparisons where significant; *d* - days; *min* - minutes; *y* - years; *g/L -* grams per liter.

A				
Population	Open	Wiltse	Percutaneous	p value
*n patients =****82***	**23**	**30**	**29**	
*n fractures =****109***	**32**	**42**	**35**	
Type A1 fractures	6 (18.8%)	11 (26.2%)	6 (17.1%)	ns
Type A2 fractures	1 (3.1%)	0	2 (5.7%)	ns
Type A3 fractures	17 (53.1%)∗	3 (7.1%)∗^	16 (45.7%)^	**∗^0.0001**
Type A4 fractures	3 (9.4%)	6 (14.3%)	1 (2.9%)	ns
Type B fractures	5 (15.6%)∗	22∗ (52.4%)∗^	10 (28.6%)^	**∗0.0018 ^0.006**
Multi-level fractures	8 (25%)∗	11 (25%)^	3∗^ (10.3%)	**∗0.04 ^0.03**
Age	39.7 ± 14.8 y	42.9 ± 19.8 y	47.1 ± 19.1 y	ns
Female sex	9 (39.1%)	11 (36.6%)	6 (20.1%)	ns
Most fractured vertebra	L1	L1	T12	
Most fractured segment	T12-L1	T12-L1	T12-L1	

B				
Outcomes	Open	Wiltse	Percutaneous	p value
Intervention delay	0.95 ± 0.95 d∗^	3.1 ± 2.2 d∗	3.6 ± 2.4 d^	**<0.001∗^**
Operating time	155 ± 52 min	201 ± 87 min	166 ± 73 min	ns
SpineJack Kyphoplasty	0	20 (66.6%)	9 (31%)	**0.009**
Second-stage corpectomy	11 (47.8%)∗^	1 (3.3%)∗#	10 (33.3%)^#	**∗0.0002 ^0.1 #0.017**
Screw system of choice	USS Fracture	USS Fracture	CD-Horizon, Viper Prime	
Preoperative pain VAS	3.8 ± 2.8/10	5.2 ± 2.7/10	5 ± 2.1/10	ns
Postoperative pain VAS	3.8 ± 1.2/10	3.2 ± 1.9/10	3.5 ± 2.1/10	ns
Discharge pain VAS	2 ± 1.8/10	1.8 ± 1.5/10	2 ± 1.7/10	ns
Length of stay	21.6 ± 10 d∗^	11.2 ± 4.5 d∗	16.5 ± 7.8 d^	**<0.001∗; 0.04^**
Rehabilitation	11 (47.8%)	13 (43.3%)	13 (41.4%)	ns
Rehabilitation duration	25 ± 12.5 d	20.3 ± 16.3 d	18.7 ± 7.2 d	ns
Functional recovery	72 ± 48 d∗	34 ± 12 d∗	57 ± 23 d	**0.002∗**
Perioperative complication	3 (13%)	2 (6%)	3 (10.3%)	ns
Surgical site infection	1 (4.3%)	1 (3.3%)	0	ns
Major long term complication	2 (8.6%)	2 (6.6%)	3 (10.3%)	ns
Minor long term complication	6 (26.1%)	8 (26.6%)	5 (17.2%)	ns
Hemoglobin loss	37 g/L∗^	25 g/L∗	17 g/L^	**0.014∗; <0.001^**
Estimated blood loss	1584 ± 1027 ml∗^	929.5 ± 453.9 ml	653.5 ± 429.8 ml	**0.003∗; <0.0001^**
Loss of correction	1 (3.1%)	1 (2.4%)	1 (2.9%)	ns

Length of stay was selected *a priori* as the primary outcome because it represents a composite real-world indicator of perioperative morbidity, recovery kinetics, and healthcare resource utilisation, and is consistently reported across retrospective trauma cohorts with minimal missing data (Lan et al., 2017).

### 3.2 Baseline cohort characteristics

There were no significant differences between groups in terms of age (Open: 39.7 ± 14.8 years, Wiltse: 42.9 ± 19.8 years, Percutaneous: 47.1 ± 19.1 years), gender distribution, or the most frequently fractured vertebral segment, which was predominantly T12-L1 across all patients (Table 1A, Fig. 2A–B). This apparent clustering at the thoracolumbar junction followed a near-normal distribution with moderate-to-excellent Gaussian fit (Fig. 2B, light blue shading). There was no difference in the distribution of A1, A2 and A4 fractures across the three cohorts. A lower proportion of A3 fractures were treated by the Wiltse (7.1%) approach compared to the Open (53.1%) and Percutaneous (45.7%) approaches (both *p<*0.0001). Conversely, a significantly higher prevalence of type B fractures were treated by the Wiltse approach (52.4%) compared to the open (15.6%, *p=*0.0018) and percutaneous (28.6%, *p=*0.006) cohorts. Additionally, multi-level fractures were less common in the percutaneous group (10.3%) compared to the open and Wiltse groups (both 25%; *p* = 0.04 and 0.03, respectively). Baseline cohort characteristics were thus largely comparable across the three groups.

### 3.3 Surgical metrics, length of stay and functional recovery

**Fig. 3:**
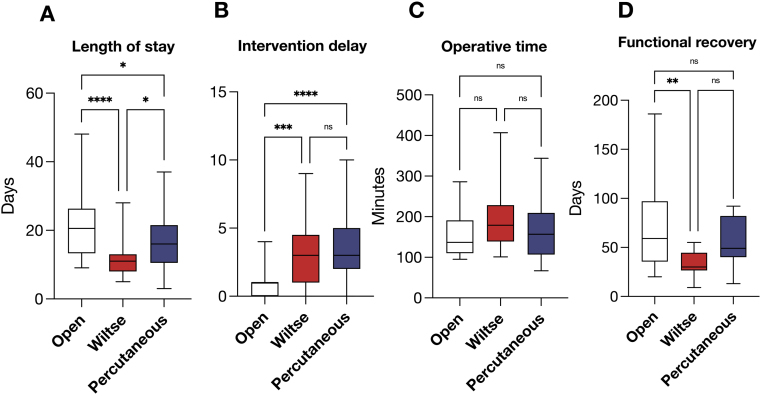
Time metrics. **A** – Length of stay. **B** - Intervention delay. **C** - Operative time. **D** - Functional recovery/return-to-work status. ∗*p <* 0.05; ∗∗*p* < 0.01; ∗∗∗*p < 0.001; ∗∗∗∗p <* 0.0001; ns - non significant.

The delay of admission-to-surgery (Fig. 3B) was significantly shorter in the open cohort (0.95 ± 0.95 days, white) compared to the Wiltse (3.1 ± 2.2 days, red) and percutaneous (3.6 ± 2.4 days, blue, *p* < 0.001). All patients in all three groups received a diagnostic workup by high-resolution CT or standard radiograph. All patients underwent post-op and follow up imaging by standing radiograph. Operative time (Fig. 3C) did not differ significantly among groups (Open: 155 ± 52 min, Wiltse: 201 ± 87 min, Percutaneous: 166 ± 73 min). As expected of our strategy, SpineJack kyphoplasty was employed significantly more in the Wiltse cohort (Table 1, 66.6%) compared to the Percutaneous (31%; p = 0.009), with no instances in the Open cohort. Conversely, a higher proportion of patients underwent second-stage surgery by vertebral corpectomy and cage in the Open (47.8%, *p<*0.001) and Percutaneous (33.3%, *p* = 0.017) compared to the Wiltse cohort (3.3%). There was no difference in corpectomy rates between the Open and percutaneous groups (*p* = 0.1). The fixation system of choice was USS® Fracture (DePuy Synthes) for patients treated via the Open and Wiltse approach, whereas the CD-Horizon® and Expedium® systems were employed in patients operated percutaneously.

Length of stay (Fig. 3A) was significantly shorter in both the Wiltse (11.2 ± 4.5 days, *p* < 0.001, red) and Percutaneous cohorts (16.5 ± 7.8 days, *p* = 0.04, blue) compared to patients treated by open surgery (21.6 ± 10 days, white). The Wiltse sample featured the overall shortest hospitalisation for MIS approaches (*p* = 0.02 for Wiltse vs. Percutaneous), suggesting that it was associated with a quicker postoperative recovery and discharge. Prevalence of rehabilitation and its duration were similar across groups, with no statistically significant differences. Functional recovery (Fig. 3D), quantified by clinically documented return-to-work status, was significantly faster in Wiltse (34 ± 12 *d*; p < 0.01) compared to Open patients (72 ± 48 *d*), with only a trend toward decrease in the Percutaneous cohort (57 ± 23 *d*). Interpretation of these findings should remain cautious, as return-to-work status reflects multiple socioeconomic and contextual factors and does not substitute for validated patient-reported outcome measures (PROMS). Perioperative complications, surgical site infections, and long-term complications (both major and minor) showed no significant differences among the approaches (Table 1).

### 3.4 Pain management

**Fig. 4:**
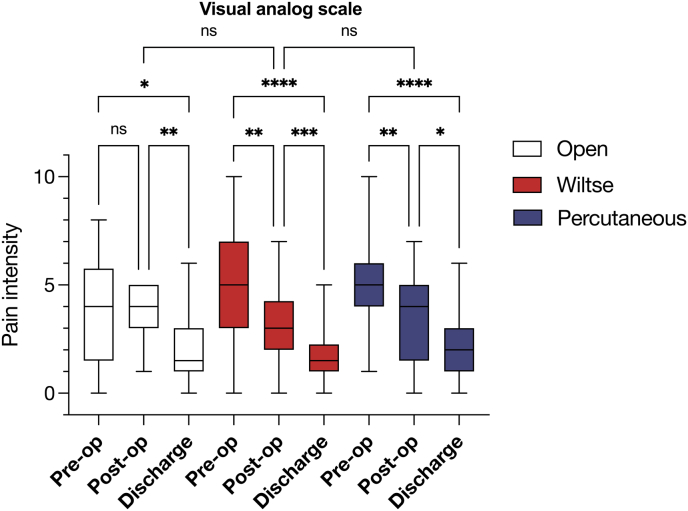
Peri-operative pain levels by visual analog scale (VAS) 0-10/10.

There was no difference in pain management strategy across all cohorts. Pain intensity was measured using a Visual Analog Scale (VAS, Fig. 4) at three time points: preoperative, postoperative at 24-48h, and at discharge. In the Wiltse and Percutaneous groups, there was a significant decrease in VAS scores from preoperative to immediate postoperative at 24-48 h (*p=*0.004, *p* = 0.003, respectively), and this reduction was further enhanced at discharge (*p<*0.001, *p* = 0.02, respectively), indicating effective pain management. The Open cohort on the other hand did not demonstrate statistically significant changes in pain intensity in the immediate postoperative period (*p* = 0.99), with levels improving significantly at discharge (*p* = 0.004). There was no difference in overall VAS scores between any of the three cohorts.

### 3.5 Radiological outcomes

**Fig. 5:**
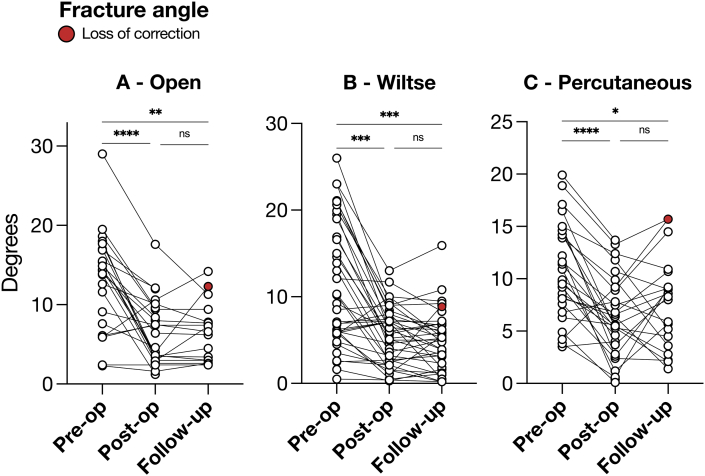
Pre-operative fracture angle correction post-operatively and at long term follow-up. Loss of correction (red) was defined as ≥ 5° increase in fracture angle at long term follow up.

One key radiographic parameter in the perioperative assessment of thoracolumbar fractures is the angle of deformation by local kyphosis. We quantified this in each cohort to determine whether the choice of approach could impact the effectiveness of surgical correction (Fig. 5). The fracture angle was evaluated at three stages: preoperative, postoperative, and at 3-to-6 months follow-up. There were no significant differences in pre-operative fracture angles amongst groups (data not shown). All three groups demonstrated a significant reduction in fracture angles from preoperative to postoperative (*p* < 0.001) and maintained this reduction throughout the follow-up period (Open: *p* = 0.001 Wiltse: *p* < 0.001, Percutaneous: *p* = 0.03). This stability indicates effective correction with minimal loss of alignment over time. Loss of correction, defined as > 5° increase in kyphotic fracture deformity at follow up, was minimal and comparable, occurring in one patient per group, with no significant differences observed.

### 3.6 Intraoperative blood loss

**Fig. 6:**
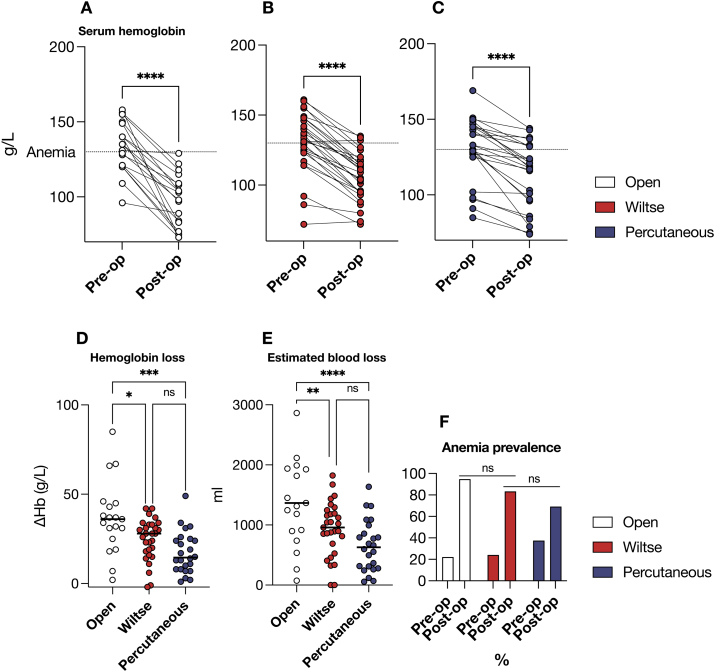
Intraoperative blood loss by peri-operative hemoglobin (Hb) levels **A, B, C -** Within-group serum Hb levels pre-versus post-operatively in the Open (white), Wiltse (Red) and Percutaneous (Blue) cohorts. **D** - Direct comparison of hemoglobin loss (ΔHb) across cohorts. **E** - Estimated blood loss (EBL) by Nadler's and Gross' formula. **F** - Prevalence of post-operative clinical anemia across the three groups.

We next quantified intraoperative blood loss using two independent estimation methods. First, we analysed the difference between pre- and postoperative haemoglobin (Hb) levels, which served as a surrogate marker of perioperative blood loss across the three cohorts (Fig. 6A–D). Second, we estimated blood loss using calculated estimated blood volume (EBV) according to the Nadler formula combined with the Gross equation based on perioperative haematocrit values (Jiang et al., 2023). The prevalence of post-operative clinical anaemia was also evaluated (Fig. 6E).

Preoperative and postoperative serum Hb levels showed a significant decrease across all three surgical approaches (Fig. 6A–C), indicating perioperative blood loss (p < 0.001 for all groups). When comparing haemoglobin loss as the difference between pre- and postoperative levels (ΔHb, Fig. 6D), the open approach showed significantly higher ΔHb compared to the Wiltse approach (37 vs. 25 g/L, p = 0.014) and the percutaneous approach (17 g/L, p = 0.0001), with no significant difference between the Wiltse and Percutaneous techniques (p = 0.1661). These findings indicate that both MIS approaches are associated with reduced haemoglobin loss compared to the Open approach.

EBV calculation (Fig. 6E) yielded 1584 ± 1027 ml in the Open group, 929.5 ± 453.9 ml in the Wiltse group, and 653.5 ± 429.8 ml in the Percutaneous group, corresponding to a significant 41.3% (655 ml) and 58.8% (931 ml) reduction in blood loss (p = 0.003 and p < 0.0001, respectively). There was no difference in the prevalence of post-operative anemia across cohorts (Fig. 6F).

## 4 Discussion

The comparison of Open, Wiltse, and Percutaneous approaches for treating neurologically intact thoracolumbar fractures reveals distinct advantages associated with MIS techniques. Our study suggests that minimally invasive approaches, particularly the Wiltse technique, are associated with favourable outcomes regarding intraoperative blood loss, hospital stay, and patient recovery.

Importantly, these findings must be interpreted cautiously given the study's retrospective design and the use of non-overlapping historical cohorts. Although institutional perioperative protocols remained stable, temporal bias cannot be excluded, as broader improvements in perioperative care, imaging technology, anaesthetic management, and rehabilitation pathways over the study interval may have influenced outcomes independently of surgical approach. Consequently, our results should be interpreted as associative rather than causal.

Length of stay was shorter in the Wiltse cohort by up to ten days compared to the open approach and up to five days compared to the percutaneous cohort. These findings overall suggest enhanced recovery associated with MIS techniques. Rapid postoperative mobilisation likely contributes to reduced hospital stays, as shown by similar studies (Junhui et al., 2017; Jiang et al., 2022; Rapp et al., 2017; Guiroy et al., 2018), which emphasise the benefits of minimised muscle dissection in preserving paraspinal musculature and reducing postoperative pain. Overall, we found our local length of stay values to be in range with existing literature for non-fusion surgery (Wang et al., 2006). Although length of stay may be influenced by institutional or social factors independent of surgical technique, we selected it as a pragmatic and reproducible endpoint reflecting overall perioperative burden. Secondary outcomes were therefore included to provide a multidimensional assessment of surgical impact.

Notably, MIS approaches significantly reduce estimated blood loss up to nearly 1 L compared to open surgery. Our experience is in line with current literature (Jiang et al., 2023; Sheng et al., 2022). It has been shown that in multilevel lumbar fusion, EBL >500 mL is an independent risk factor for prolonged stay and increased postoperative complication (Chen et al., 2023), and that perioperative blood loss is strongly correlated with intra/postoperative complications and surgical revisions (Lange et al., 2022). These findings suggest a potentially clinically meaningful reduction in blood loss, although their direct clinical impact should be interpreted cautiously. Visual estimation of intraoperative blood loss is inherently imprecise due to variability in irrigation fluid volume and suction measurements. Haemoglobin- and haematocrit-based methods therefore provide a more standardised surrogate, but they remain indirect estimates and may be influenced by perioperative fluid shifts, haemodilution, or timing of laboratory measurements. Importantly, transfusion data were not consistently available in this retrospective cohort, which limits the ability to determine the clinical significance of observed between-group differences. Accordingly, these findings should be interpreted as comparative trends rather than definitive measures of true blood loss. While the statistical differences observed between approaches were robust, statistical significance does not necessarily equate to clinical significance, and the magnitude of difference should be interpreted within the broader perioperative context.

Our findings also suggest that pain management post-MIS surgery is effective and has faster kinetics than open approaches, as evidenced by the reduced VAS scores from preoperative to discharge stages. This is also consistent with current evidence (Jiang et al., 2022; Li et al., 2015).

Radiologically, all three approaches provided stable correction of the fracture angle over the follow-up period, with minimal loss of alignment, corroborating the findings in recent studies (Junhui et al., 2017; Kerschbaumer et al., 2019; Lei et al., 2020; Lu et al., 2021) that MIS techniques can achieve similar radiological outcomes to open surgery in terms of fracture stability and alignment. However, the Wiltse approach showed the highest percentage of type B fractures treated and multi-level fractures in the two MIS cohorts, suggesting local surgeons’ preference for this technique in complex fracture patterns. This is possibly due to the more controlled visualisation and access provided. In all cohorts, most fractures treated were AO Spine A-type, including A1 in about a fifth of patients. This finding should be interpreted carefully. As a matter of fact, indication for fixation was also heavily determined by pre-operative kyphotic deformation and pain levels, not just fracture type alone. This emphasises the importance of employing composite diagnostic assessment when establishing clinical indications.

There was no difference in prevalence of A-subtypes between the cohorts, except for a significantly lower proportion of A3 fractures in the Wiltse cohort. This might reflect a preference for the percutaneous approach in this fracture type when shifting to MIS. However, the sample size might be too small to distinguish this conclusion from a pure difference in incidence at our centre.

Differences in fracture morphology between cohorts should be interpreted in the context of real-world surgical decision-making rather than as methodological imbalance. The choice of surgical approach for thoracolumbar fractures is strongly influenced by injury morphology, biomechanical stability, and involvement of the posterior ligamentous complex, as reflected in established classification systems and treatment algorithms (Schnake et al., 2017; Divi et al., 2019; Wood et al., 2014). Consequently, variations in fracture type distribution between groups likely reflect case selection and operative indication rather than intrinsic superiority of a given technique. Observed differences in outcomes such as corpectomy or augmentation rates should therefore be interpreted cautiously, as they may partly represent baseline injury complexity rather than purely technique-related effects. Similar considerations have been highlighted in comparative studies of surgical strategies for thoracolumbar trauma (Zhu et al., 2023; Jiang et al., 2022; Sheng et al., 2022).

Second-stage corpectomy was performed more frequently in the Open and Percutaneous cohorts compared to the Wiltse cohort. Conversely, SpineJack kyphoplasty® was employed only in MIS, and more frequently in the Wiltse cohort. These findings likely reflect both evolving surgical strategy and differences in baseline fracture morphology between cohorts rather than purely technique-dependent effects.

Since fixation alone may not always provide adequate biomechanical support for multilevel fractures or severe vertebral body collapse, secondary corpectomy was traditionally thought necessary to ensure optimal stability and alignment. This remains standard practice in many centres. Current literature still posits that fixation may need to be supplemented with additional procedures to achieve satisfactory outcomes in more challenging cases such as significant kyphotic deformity or burst fractures (Maciejczak et al., 2007; Pham et al., 2017). Whilst this principle remains, here we show that in the Wiltse cohort, the majority of corpectomies were replaced by vertebral augmentation, where corpectomy was only employed in one case. At present, we reserve the procedure for complex pattern fractures (e.g.: A4, complete burst), whereby simple kyphoplasty remains impractical due to the challenging architecture of bone substrate for augmentation.

Thus, as introduced, MIS approaches with concomitant SpineJack® insertion where indicated reduces overall morbidity, but also obviates the need for a staged corpectomy in most cases, effectively consolidating stabilisation and vertebral augmentation into a single procedure (Jiang et al., 2022; Sheng et al., 2022).

The integration of SpineJack kyphoplasty with MIS techniques has also shown promise in reducing hospital stay and enhancing early mobilisation (Giordan et al., 2022). Evidence shows that incorporating kyphoplasty in MIS surgeries for thoracolumbar fractures may not only improve vertebral kyphotic restoration but also contributed to lower pain scores and faster recovery (Giordan et al., 2022; Finoco et al., 2023). These results align well with our study's observation that the MIS cohort had shorter hospitalisation and effective pain management outcomes.

From a broader perspective, these findings support the notion that MIS techniques can effectively address complex thoracolumbar fractures. This trend is further corroborated by recent clinical literature (Dong et al., 2014; Jin et al., 2021), which highlights the advantages of combining MIS techniques with vertebral augmentation procedures, thereby offering a comprehensive approach to fracture management that minimises the need for secondary procedures.

Interestingly, patients operated by the open approach showed a shorter delay to intervention. One plausible explanation is that, at the time those procedures were performed (2010–2012), open surgery represented a straightforward, well-established standard of care. The assumption that second stage corpectomy would almost always be planned after initial stabilisation meant that patients could be triaged and taken to surgery more rapidly with fewer logistical requirements (e.g., no specialised navigation platforms or newer instrumentation). By contrast, the more recent MIS approaches (2021–2023) often involve advanced imaging, specialised equipment, and the availability of a team trained in these techniques, thus potentially delaying operative scheduling. Additionally, evolving institutional protocols may further explain why these newer techniques had a relatively longer intervention delay despite ultimately offering improved perioperative metrics.

Whilst our data clearly show an advantage of MIS v Open approaches, there were no significant differences in most outcomes between the Wiltse and Percutaneous cohorts, which featured similar intraoperative blood loss, pain kinetics and only a marginal reduction in hospitalisation stay in the Wiltse versus Percutaneous groups. However, we identified significant differences in patient selection between the two approaches, whereby Wiltse was employed to treat multi-level and type B fractures more frequently. Thus, we argue that whilst both MIS strategies offer comparable outcomes and advantages over open surgery, the semi-open Wiltse approach has been our choice for more complex fractures. However there is insufficient data to further assess how this technique compares to fully percutaneous fixation in this subset of fractures.

In conclusion, our data highlight key advantages of the Wiltse and Percutaneous approaches over traditional open surgery, which are achievable without sacrificing radiological and clinical outcomes.

## 5 Limitations and future directions

While this study provides valuable insights into the comparative benefits of open, Wiltse, and percutaneous approaches for thoracolumbar fractures, certain limitations should be noted. The retrospective, single-center design offers an overview of evolving practices at our institution but may not capture nuances in approach across different centres. The sample size may be too small to reliably capture differences in complication rates. As mentioned above, the use of a historical comparator cohort inherently introduces potential temporal bias. However, our institutional perioperative protocols, including early mobilisation, proactive multimodal analgesia, routine physiotherapy involvement, and rehabilitation pathways, have been longstanding and remained stable across the two periods. Table 1 shows no significant differences in rehabilitation prevalence or duration across groups. Pain management strategies did not differ, and VAS trajectories support comparable postoperative handling across cohorts. Nevertheless, we maintain that differences observed between cohorts cannot be attributed solely to surgical technique and should be interpreted as compounded by associative rather than purely causal effects.

One potential source of bias might lie in differential surgeon experience. This was at least partly controlled by the single-centre design, as all procedures being performed within the same departmental structure, under a stable institutional protocol, and in most cases by the same senior surgical team across both surgical approaches.

As mentioned above, this study lacks validated PROMS such as the ODI or SF-36, which represents an important limitation. These instruments were not routinely collected for urgent trauma cases during the study periods, and retrospective acquisition is not feasible. Consequently, functional recovery could only be assessed using clinically documented variables such as return-to-work status. While pragmatic and consistently available, this measure is an imperfect surrogate for patient-perceived disability and may be influenced by social, occupational, or administrative factors. Therefore, our functional outcome findings should be interpreted cautiously and primarily as exploratory indicators rather than definitive assessments of patient-reported recovery.

Future research can build on these findings by exploring the long-term benefits of MIS techniques across broader patient populations and surgical teams. Expanding the follow-up period would allow for a more comprehensive assessment of long-term outcomes, including alignment maintenance, complication rates, and patient satisfaction. Additionally, advancements in image-guided technology and MIS instrumentation promise to further improve precision and applicability in complex fractures, reducing the need for secondary procedures. When secondary procedures are inevitable, emerging MIS technologies such as robotic assistance may further expand the precision, reproducibility, and applicability of minimally invasive spinal approaches, representing an important avenue for future investigation (Choucha et al., 2025). These efforts can help refine surgical protocols and optimise patient outcomes, particularly as minimally invasive techniques continue to expand and evolve in thoracolumbar fracture management.

## Declaration of contributions

SG conceived the study, collected and analysed the data, wrote the first draft of the paper, designed the figures, edited the final draft and acted as assistant surgeon. BV collected and analysed the data, wrote first draft of the paper and edited the final draft. FB collected the data and edited the final draft. EC collected the data. AM conceived the study, critically assessed the data, edited the drafts of the paper and acted as principal or assistant surgeon. GM, NL and DD critically assessed the data, edited the final draft of the paper and acted as principal surgeon. KS critically assessed the data edited the final draft of the paper. ET supervised all authors, conceived the study, critically assessed, edited all drafts of the paper and acted as principal surgeon. All principal surgeons participated in local adoption of the surgical approaches and introduction of the vertebral augmentation.

## Declaration of competing interest

The authors declare the following financial interests/personal relationships which may be considered as potential competing interests:Enrico Tessitore reports a relationship with Spineart Geneva SA that includes: consulting or advisory. Enrico Tessitore reports a relationship with Corporate Brainlab AG that includes: consulting or advisory. Enrico Tessitore reports a relationship with Medability that includes: consulting or advisory. If there are other authors, they declare that they have no known competing financial interests or personal relationships that could have appeared to influence the work reported in this paper.
